# Venolymphatic Malformation Presenting as a Low-Flow Vascular Anomaly: A Case Report

**DOI:** 10.7759/cureus.103061

**Published:** 2026-02-05

**Authors:** Gavara Yogyasri, K Saivenkatamuni Krishna, Nisarga G, Thumbichetty Girish, Chandrashekar D

**Affiliations:** 1 Department of General Surgery, Vydehi Institute of Medical Sciences and Research Centre, Bengaluru, IND

**Keywords:** congenital abnormalities, head & neck trauma, image-guided sclerotherapy, low-flow vascular anomaly, venolymphatic malformations

## Abstract

Venolymphatic malformations (VLMs) are rare congenital disorders involving the vascular and lymphatic systems. They are characterized by altered endothelial differentiation rather than true neoplastic proliferation, with lesion enlargement occurring in response to hormonal or inflammatory stimuli. We report a case of a 15-year-old male who presented with slow-growing swelling over the right parotid region that worsens with trauma, requiring multidisciplinary care. Clinical examinations and imaging findings on ultrasound and magnetic resonance imaging were suggestive of VLM, a low-flow venous malformation, and a cystic epidermoid lesion of the right parotid gland. The patient went under image-guided sclerotherapy under interventional radiology guidance. This case highlights the importance of multimodal imaging in establishing the diagnosis of VLM and demonstrates the role of sclerotherapy as an effective minimally invasive treatment option for selected head and neck swellings.

## Introduction

Venolymphatic malformation (VLM) is a rare congenital disorder that involves both the venous and lymphatic channels [1]. The International Society of Vascular Anomalies (ISSVA) classification provides a basis to differentiate between various malformations, according to which VLM is a combined vascular malformation [1]. This classification aids in guiding direct treatment methods such as topical 1% sirolimus cream [2]. According to blood flow characteristics and the type of vessel involved, vascular malformations are divided into low-flow and high-flow malformations [3]. Low-flow malformations include venous, capillary, lymphatic, and combined vascular malformations, whereas high-flow malformations are characterized by arterial and arteriovenous involvement [3]. VLM is a combined low-flow vascular malformation and results from persistent abnormal development of venous and lymphatic channels, which progress with age [4]. This congenital disorder affects both genders and commonly involves the head and neck region, with abdominal involvement being a rare presentation [4]. Some swellings may be present as uniseptate or multiseptate, and their postnatal management depends on the size and location of the swelling [5]. Treatment options range from surgical debulking to other minimally invasive options such as sclerotherapy, embolization, and cryoablation, which are often employed as a part of a multimodal management strategy [6]. In certain cases, these swellings can also be life-threatening and may be present since birth with symptoms such as pain, bleeding, or cosmetic disfigurement [3]. It can also affect vital organs during infancy, childhood, or adulthood [3]. Magnetic resonance imaging (MRI) plays a pivotal role in the diagnosis of VLM by aiding in the differentiation between uniseptate and multiseptate swellings, thereby facilitating more accurate diagnosis and improved clinical management [7]. Further studies may contribute to a better understanding and optimization of treatment strategies for this condition.

## Case presentation

A 15-year-old male patient presented to the general surgery outpatient department with complaints of swelling on the right side of his face since birth, which was initially small, gradually increasing in size. The patient underwent fine-needle aspiration cytology (FNAC) five months back, elsewhere, following which the swelling was noted to have progressively increased in size. It is aggravated mainly during winter, associated with headaches. The child was a full-term baby with an unremarkable perinatal history, achieved age-appropriate milestones, and was immunized as per the national schedule. There was no significant family or social history. 

On general examination, anthropometry and vital signs were within normal limits (Table 1). The patient was afebrile with no signs of pallor, icterus, cyanosis, clubbing, or lymphadenopathy.

**Table 1:** Baseline vital signs

Parameter	Patient value	Reference range
Blood pressure	110/70 mmHg	120/80 mmHg
Pulse rate	78 beats/min	60-100 beats/min
Temperature	36.8ᵒC	36.5ᵒC-37.5ᵒC
Respiratory rate	16 breaths/min	12-20 breaths/min
Oxygen saturation	98%	95%-100%

**Figure 1:**
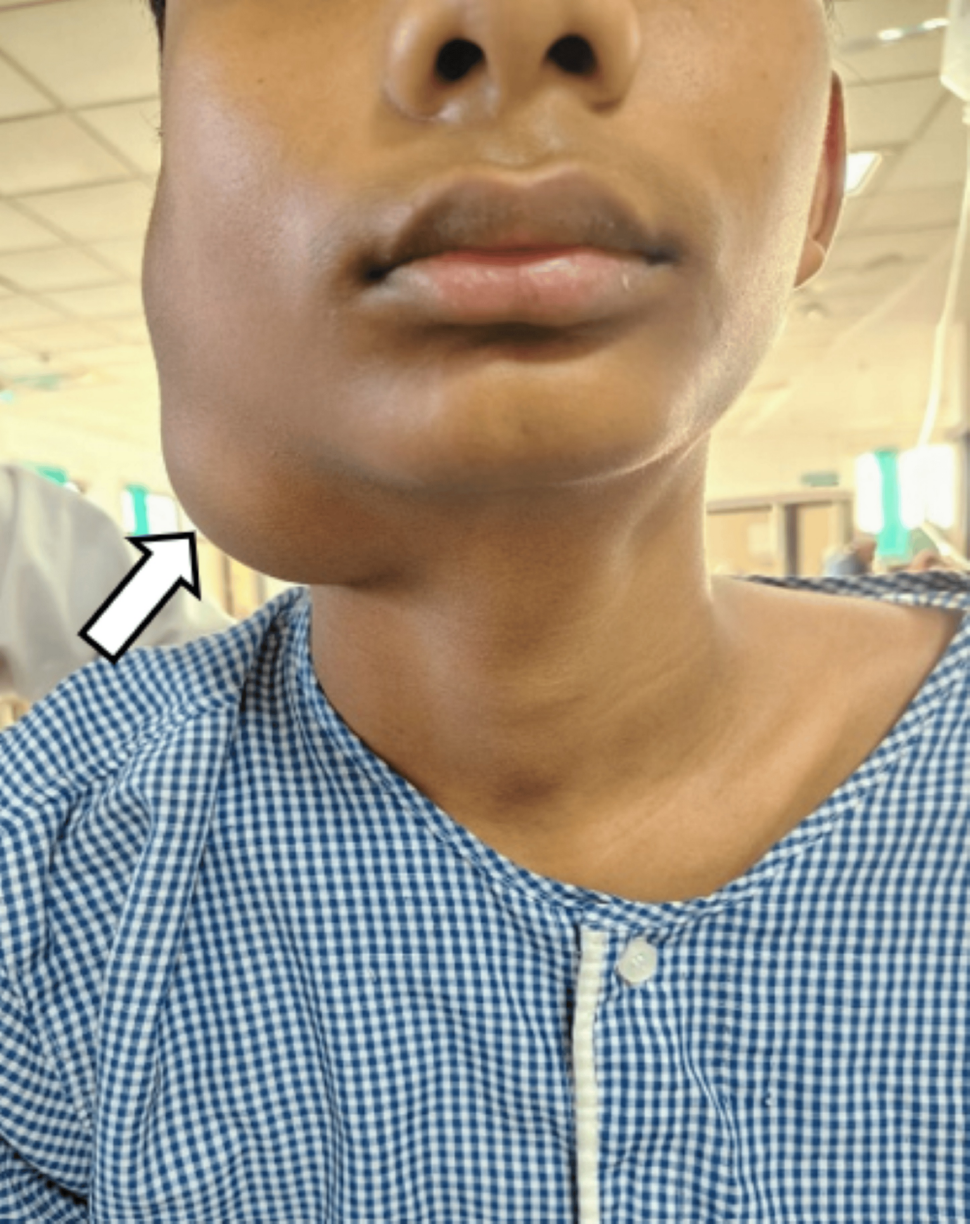
Clinical photograph showing swelling over the pre-auricular and infra-auricular region extending into the neck.

On further examination of the patient, a solitary ovoid swelling measuring approximately 6 x 4 cm was noted over the right infra-auricular region, extending 2 cm inferior to the angle of the mandible, laterally up to 2 cm anterior to the mastoid process, and medially 1 cm posterior to the coronoid process of the mandible, and upward displacement of the ear lobule. The surface of the swelling is smooth with a well-defined margin. The skin over the swelling was normal, with no discharge from the swelling and no visible pulsations or venous engorgements over the swelling (Figure 1).

**Figure 2:**
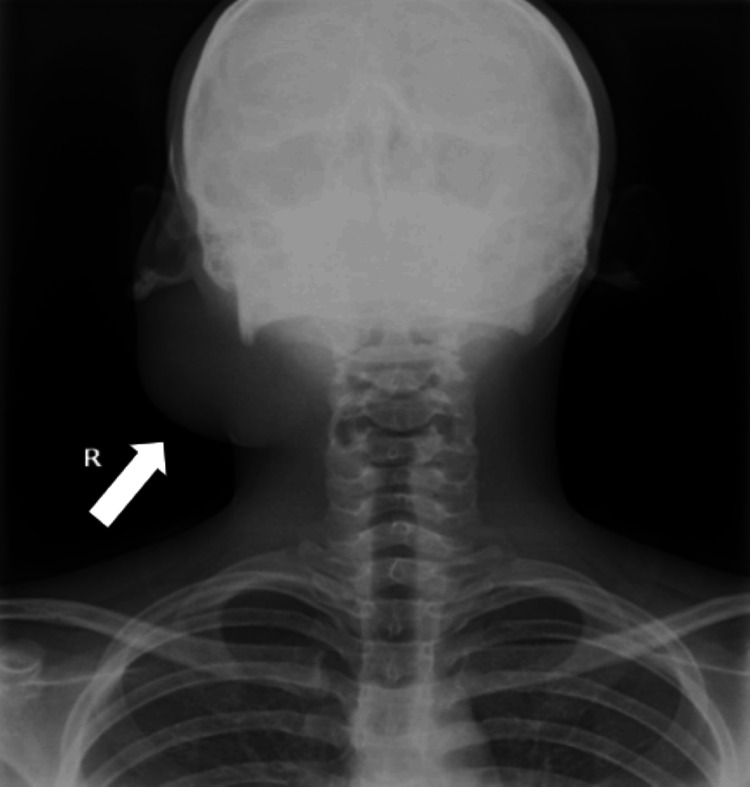
X-ray of the patient showing the swelling near the right infra-auricular region.

The swelling was soft in consistency, non-tender, mobile, and not fixed to any underlying structures. The surface of the swelling is smooth and pinchable, with no palpable cervical lymph nodes, no enlargement of the deep part of the parotid gland, no discharge from the parotid duct, and normal facial muscles function. Examination of the other salivary glands was normal. X-ray of the patient in the anteroposterior view showing soft tissue swelling with no internal calcifications (as phleboliths are the only significant radiographic finding in VLM on X-ray) (Figure 2).

**Figure 3:**
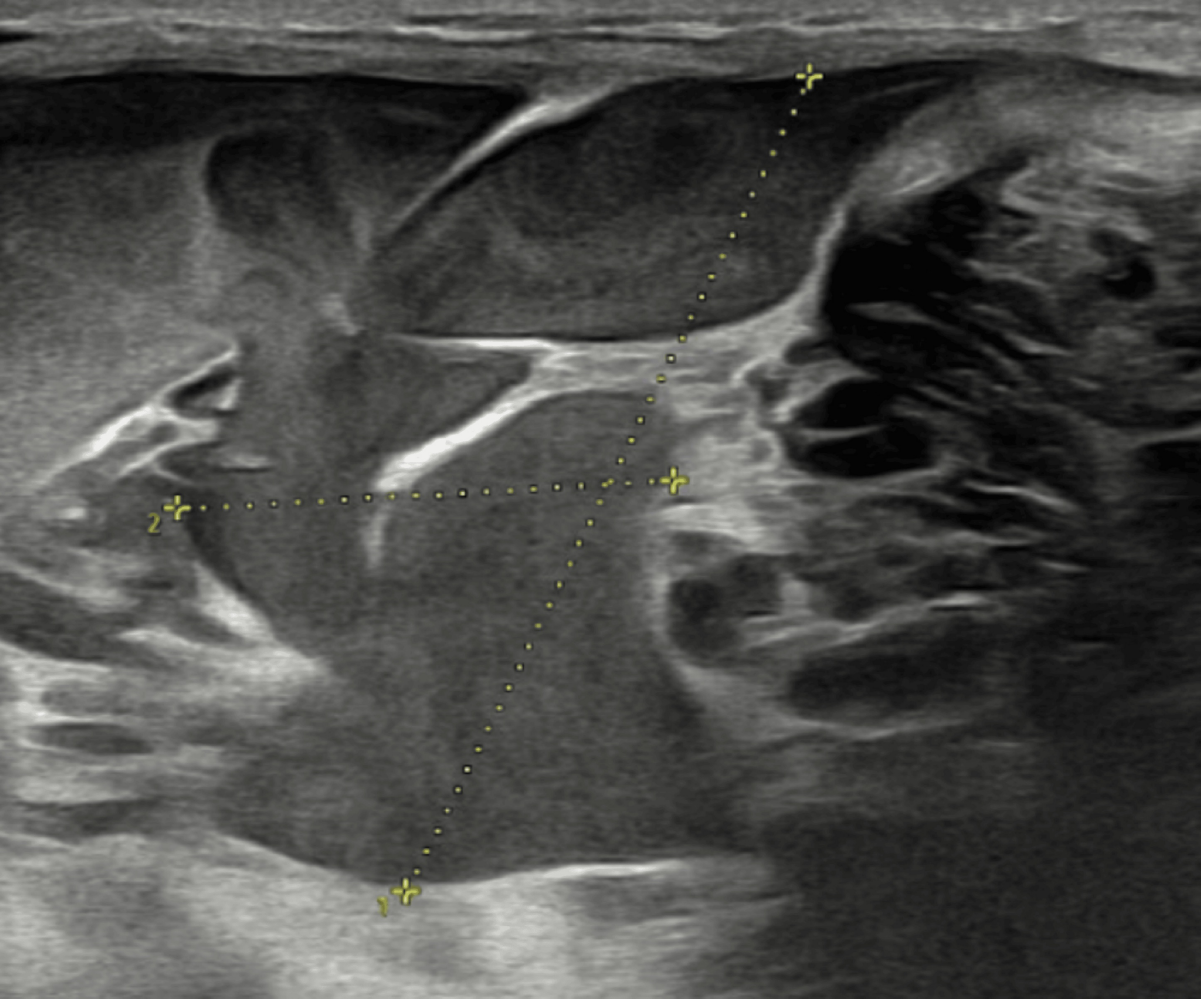
Ultrasonography of the right parotid region demonstrating a cystic lesion consistent with venolymphatic malformation.

Ultrasound of the right parotid region revealed a large, well-defined heterogeneously hypoechoic area measuring approximately 83 x 42 x 20 mm (volume: 35-40 cc), involving the right superficial and deep parotid glands with multiple variable-sized cystic lesions communicating with each other, and showing pultaceous mobile internal contents. Normal color flow was noted on Doppler interrogation. Based on the ultrasonographic features, the differential diagnoses included a VLM and epidermoid cysts. Although epidermoid cysts are typically avascular, they were considered due to cystic appearance on grayscale imaging; however, the presence of internal septations and vascularity on Doppler favored a VLM over epidermoid cyst (Figure 3).

**Figure 4:**
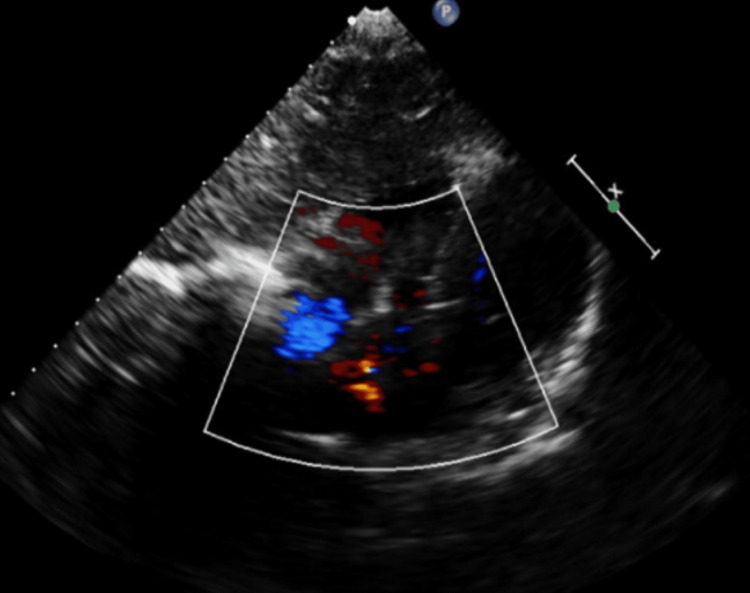
Color Doppler ultrasonography of the right parotid region demonstrating low-flow vascularity consistent with a venolymphatic malformation.

Ultrasonography Doppler as a part of further investigation was performed (Figure 4).

**Figure 5:**
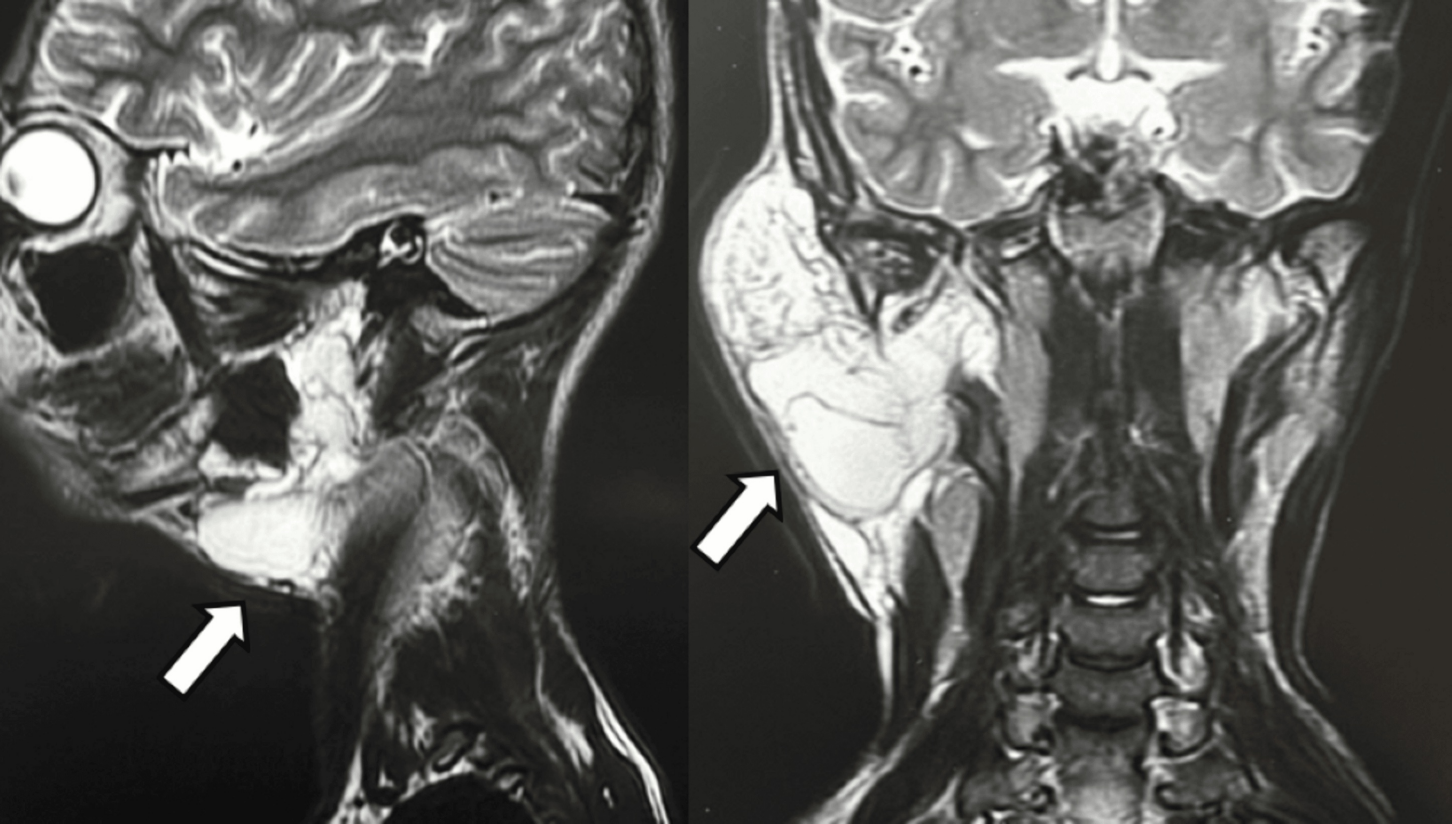
MRI showing the multiloculated, multiseptated lesion in the right infra-auricular region. MRI, magnetic resonance imaging.

MRI showed an altered signal-intensity lesion (hyperintense on short tau inversion recovery) involving the superficial and deep lobes of the right parotid gland. The right parotid parenchyma was not visualized separately. The lesion showed multiple thin-to-thick internal septations, likely representing intracystic hemorrhage. There was an extension into the submandibular space with a mass effect on the ipsilateral submandibular gland, causing displacement anteromedially. These findings were consistent with the diagnosis of VLM (Figure 5).

The clinical behavior, together with ultrasonographic findings, including venous and arterial Doppler evaluation, favored a diagnosis of VLM. The MRI findings played a key role in the diagnosis.

In view of the above investigations, sclerotherapy was planned after discussion of available treatment options with the patient and guardian, and informed consent was obtained. The procedure was performed under local anesthesia with strict aseptic precautions, using bleomycin as the sclerosing agent, administered intralesionally at an approximate dose of 0.5 mg/kg body weight in a single session. Bleomycin was preferred due to its efficacy and favorable safety in low-flow VLMs, particularly due to its predominant action on the lymphatic component, as seen in our patient. The patient remained hemodynamically stable and was discharged in stable condition. On follow-up, a reduction in the size of swelling was noted, and the patient was advised to undergo outpatient review after three months. 

## Discussion

According to the ISSVA classification, VLMs are combined vascular malformations involving both venous and lymphatic components [1]. This case is rare since VLM is a relatively uncommon condition, with an incidence of 1%-1.5% of the population [3], and occurs on a scale of 1.2 to 2.8 per 1,000 newborns [4]. Identification of vascular malformations can be challenging and can often be confused with vascular proliferations. The distinction between them is based on histopathological studies of increased cell turnover [8]. Vascular proliferations are true neoplasms characterized by rapid cell growth in postnatal life and show slow regression into late childhood [8]. In contrast, vascular malformations are abnormally formed channels within the vascular apparatus, lined by endothelial cells that do not undergo rapid cell growth. They often go unnoticed at birth and progress with growing age [8].

Given the wide anatomical distribution and heterogeneous nature of VLMs, imaging plays a crucial role in establishing an accurate diagnosis [9]. Doppler ultrasonography and MRI helped to exclude other differential diagnoses such as branchial cyst (unilocular, simple cyst with no venous channels involved) [10], hemangiomas (high-flow, voids and intense enhancement) [11], and cystic hygroma (purely lymphatic with no venous channel involvement) [12]. The imaging findings in the patient demonstrated features consistent with both venous and lymphatic components, supporting the diagnosis of VLM rather than an isolated venous or lymphatic lesion. In the present case, invasive diagnostic procedures such as biopsy or FNAC were not performed; the imaging findings were considered sufficient to establish the diagnosis, and the vascular nature of the swelling was taken into account. Clinically, such lesions may show changes in size or consistency with factors such as temperature, which can raise a suspicion of VLM as occurred in the patient. Given these considerations, imaging plays a central role in diagnosis [9]. In cases of parotid swelling demonstrating low-flow vascular characteristics, referral to an appropriate specialist is advisable for further evaluation and management.

Given their clinical variability, accurate diagnosis and appropriate treatment play a key role in patient outcomes. Sclerotherapy is considered the first line of treatment, with bleomycin commonly preferred as the sclerosing agent [7]. In the present case, the patient underwent the same procedure and showed signs of improvement. Although bleomycin is the preferred treatment, there are relatively few studies demonstrating its effectiveness [7]. Percutaneous sclerotherapy constitutes a widely used treatment approach, employing various sclerosants such as bleomycin, ethanolamine oleate, and doxycycline [13]. Surgical excisions remain an option in selected cases, particularly when the swellings are deep-seated, including those involving the parapharyngeal space [14]. In such situations, robotic oral surgery has been shown to be beneficial by improving surgical access and precision [14]. Despite advances in both surgical and sclerotherapeutic techniques, there remains ongoing debate regarding the optimal primary treatment modality, and robust evidence demonstrating complete cure remains limited [7,14].

## Conclusions

VLMs represent rare low-flow vascular anomalies that pose significant diagnostic and therapeutic challenges due to their variable clinical presentation and anatomical extent. This case highlights the critical role of detailed clinical evaluation combined with multimodal imaging, Doppler, ultrasonography, and MRI in establishing an accurate diagnosis and guiding management without the need for invasive diagnostic procedures. The favorable response to sclerotherapy in our patient supports its role as an effective and minimally invasive option for selected low-flow swellings. However, given the wide variability in swelling composition, location, and clinical behavior, management must be individualized based on clinical and radiological findings. Optimal management requires further clarification; however, this case report shows a practical diagnostic approach toward a successful therapeutic outcome in a rare case presentation of VLM.
